# Karnes County Medical Society

**Published:** 1904-08

**Authors:** W. A. King

**Affiliations:** Secretary


KARNES COUNTY MEDICAL SOCIETY.—The Karnes County Medical Society met in Runge, Texas, July 11th. Meeting called to order by President Dr. W. S. Picket. The following members were present: Dr Simmons, Charco; Dr. Hamock, Choat; Dr. J. Woolsey, Riddleville; Dr. Theodore Bauhring, Nordheim; Dr. Moore, Runge; Dr. Weilburn, Runge; Dr. Robinson, Runge; Dr. M. Forbes, Kenedy, Dr. W. S. Picket, Karnes City; Dr. W. A. King, Falls City.

Dr. J. Woolsey, Riddleville, had a very interesting case before the society. Dr. W. S. Picket of Karnes City had a paper on "Cholera Infantum," which was very favorably received. Dr. S. S. Robinson of Runge read a paper on "The Responsibility of the Physician in Obstetrical Work."

The society is in a flourishing condition and is considered to be the best organization of its kind in South Texas.

W. A. KING, Secretary.

